# Somnoforme

**Published:** 1904-04

**Authors:** 


### Reviews of Dental Literature.

Somnoforme.[1] By W. H. Gilmour, L.D.S. (Eng.).

Dr. Rolland, Dean of the Dental School of Bordeaux, in his capacity of Professor of Anæsthesia, not feeling satisfied with the anæsthetics in general use for dental purposes, made up his mind to try, if possible, to formulate or discover an anæsthetic that would be more idealistic from a dental point of view.

He first of all resolved his problem under three headings or rules,—viz.:

1. In order that anæsthesia should be produced, the tension of the anæsthetic vapor should be superior to that of oxygen and to replace it in a certain quantity in the blood.

2. That the more a vapor or gas is volatile the greater in con-

[1] Paper read before the Liverpool District Odontological Society, March 17, 1903.

sequence will be its tension and the more easy its substitution for oxygen.

3. That the ideal anæsthetic would be one that approaches most nearly the conditions of absorption and elimination of oxygen in the blood.

Since most anæsthetics are administered by the respiratory organs, he therefore calculated the amount of air-space in the lungs, tabulating the amount of air taken in by an ordinary inspiration, the amount of residual, complimentary, and reserve air, and, in order to arrive at the necessary volume of anæsthetic vapor for each dose, he then calculated the tension of oxygen in the lungs and determined two further rules for the absorption of his ideal anæsthetic,—viz.:

That the greater the tension of a gas in the pulmonary vesicules, the more easily will be its absorption.

That the volatility of a gas determining its pressure, it follows that the more volatile a gas, the greater the rapidity of its absorption by the blood.

The lungs being the means of entry of the air or a vapor into the system, the blood is the distributer through its red corpuscles.

The amount of blood in the human system was then calculated, and the length of time it took to complete the cardiac circle was determined,—viz., twenty-five minutes or, more simply, thirty minutes. In other words a red blood-corpuscle would occupy thirty minutes from the time it left the heart to return to it again.

Knowing further that oxygen is only active in the arterial blood and passive in the pulmonary, it follows by simple division that oxygen is only active during fifteen minutes.

The ideal anæsthetic, therefore, being absorbed under the same conditions as oxygen, ought or will produce its effect in fifteen minutes; again it follows that if the doses are not renewed the anæsthetic ought to eliminate itself, measure by measure, as the red corpuscles return to contact with oxygen or air. It is in this manner that Dr. Rolland states that somnoforme acts.

Somnoforme is the name given to the mixture composed of chloride of ethyl, sixty per cent.; chloride of methyl, thirty-five per cent.; and chloride of ethyl, five per cent.

As to the action of an anæsthetic on the nervous system very little is known, but Dr. Rolland states that somnoforme excites the great sympathetic, producing increased cardiac contraction and arterial tension, proved by sphygmographic tracings of his own radial artery during anæsthesia, but as to how this takes place he does not know, but hopes some day to be able to find the key.

Somnoforme is sprayed into a funnel-shaped mask (formed out of a handkerchief with a sheet of parchment paper placed between the folds [1]) which is placed over the respiratory passages, care being taken to allow of no admission of air or escape of vapor.

*Signs of Anæsthesia.*—The patient must be requested to keep the eyes open during administration. When anæsthesia is about complete the eyelids will fall or the eyeballs become fixed.

The patient may also be asked to hold up the left arm, which will in time either assume a fixed or cataleptic position or fall to the side relaxed.

The patient briefly and rapidly passes through the following stages: Subconsciousness, noticing things that are going on, hearing words, sensible to touch and pain; analgesic condition, insensible to pain; anæsthesia absolute. The stages are naturally varied by the idiosyncrasies of the patient.

Whilst reflexes may be present one can still operate. With a dose of five cubic centimetres of the drug, fifty seconds to five minutes anæsthesia can be produced.

The patients recover in slightly modified ways, which can soon be learnt, the complete recovery being usually preceded by several deep inspirations. To prolong the anæsthesia it is simply necessary to replace the mask with another dose from time to time on the patient showing signs of recovery.

*Clinical Observations.*—Dr. Bailey and myself have administered somnoforme to one hundred patients at the Dental Hospital, giving an average length for administration from one dose (five cubic centimetres) of thirty-eight seconds, and an average length of fifty-one and one-sixth seconds complete anæsthesia.

In my own cases the average length of administration was thirty-nine and one-half seconds, giving fifty-five and one-sixth seconds anæsthesia. Our best results are as follows: fifteen seconds giving fifty seconds anæsthesia; twenty seconds giving one minute; twenty-seven seconds giving one minute twenty seconds; thirty seconds giving one minute thirty seconds; fifty seconds giving one

---

[1] Dr. Field Robinson, of Bordeaux, has since brought out an ideal mask which insures more accurate and uniform results.

minute forty seconds; thirty-five seconds giving two minutes five seconds.

These statistics, like all others, may or may not be very reliable as a guide; roughly speaking, I should say twenty-five to thirty seconds for administration giving an average duration of anæsthesia from fifty to sixty seconds.

Most of the cases are recorded from hospital patients, and we all know what peculiar creatures they are; one never obtains the same satisfactory results with them. Apart from the fact that they discuss the gruesome details whilst collected together in the waiting-room, recipients worthy or otherwise of the benefits of a charity are never thoroughly responsive, having an ingrained conviction that they are always being experimented upon, consequently retaining very little confidence in the operation inducing extreme nervousness.

In private practice most of these adverse conditions are absent, the results therefore being much more satisfactory.

For the single dose (five cubic centimetres) administrations we selected only those cases were it was necessary to extract a few teeth; the average number of teeth and roots extracted being from four to five.

In one case only have we failed to extract a tooth, and he was a pensioned-off soldier from South Africa, of alcoholic habits, extremely strong and excitable, although even to him we had a week previously administered the drug with success.

In a great number of our single dose administrations, seven teeth were extracted, our greatest number being thirteen.

Our average would, undoubtedly, have been greater had we been more courageous in our primary cases, and had the faith in the anæsthetic which we ultimately acquired.

We had a very fair number of male patients, all giving equally as satisfactory results as the females. One patient only refused to take the anæsthetic after commencing the administration, and she ripped the mask off her face, her reason being that she thought we were giving her chloroform, and as she had been a waitress in a dentist's house, and in the habit of attending to patients during the administration of anæsthetics, she was consequently more nervous.

Difficulty was experienced in administering the drug to the highly nervous, and also the alcoholic patients, though certainly not so great as with other anæsthetics, owing probably to the greater rapidity of anæsthetization. There was a marked quietness shown by nearly all patients during anæsthesia, particularly when compared to that of nitrous oxide. In most cases there was no period of excitation, and in all cases a complete absence of jactitation and stertor, very little if any change of color taking place, and then only by obstruction to breathing, sometimes by the blood in the pharynx, or by depression of the lower jaw when extracting lower teeth.

The breathing after one or two nervous inspirations (and this can be checked by asking the patient to swallow) becomes quiet and regular, resembling the normal breathing during sleep. We have remarked a particularly advantageous condition of analgesia which allows of the operation being continued until within a second or so of complete recovery, no patient ever showing signs of that condition of hypersensitiveness so often observed just before recovery from nitrous oxide anæsthesia. None of the patients showed any signs of retching or vomiting. The tongue retained its natural size throughout, and never abnormally interfered with the extraction of lower roots or teeth, another distinct advantage over nitrous oxide.

The bleeding was much the same as would occur after an extraction under normal conditions without an anæsthetic.

*Signs of Anæsthesia.*—We have not found the drooping of the eyelid or the fixity of the eye to be so prevalent a sign as suggested by Dr. Rolland, or even when present so accurate as the rigid or cataleptic position of the upheld arm or its other condition of relaxation. One most prevalent sign has been the cataleptic condition (this I may say in no way interferes with the operator) ; it is also with patients in this condition that our best results have been obtained. The conjunctival reflex was not always lost in commencing, and in no case have we lost the corneal. The other reflexes we have not yet been able to ascertain through want of the necessary time and opportunity to test. The pulse in most cases at the commencement is rapid on account of the patient's either expressed or hidden nervousness. It, however, in a short time assumes a regular, full, strong beat, which persists during the whole period of anæsthesia. In several cases I was conscious of a slight lowering of the pulse after the first or second inspiration, due, I imagine, to the probable shock from the cold volatile vapor. The breathing also assumed a very regular type, so much so that Dr.

Bailey thought he could take it as a definite sign of complete anæsthesia.

The recovery of all the patients was very rapid, the patient recovering his or her normal condition at once, and being able within a second or two to walk away without signs of giddiness and without help.

In one case only was there any sign of collapse, and she explained afterwards that she was subject to frequent fainting attacks.

With the exception of the last patient we have had only two who complained of any after-effects, and then only for a few seconds. In one case the patient complained of earache, and the other of headache. Against this, again, I had one patient in private practice who complained of violent headache before administration, and who, on recovering from anæsthesia, was delighted to find that her headache had disappeared as well as her teeth.

We have lately prolonged the anæsthesia by replacing the mask (into which a second dose has been sprayed) on the patient showing signs of recovering, and in this way the patient has remained anæsthetized during a period of three and one-half and three and one-quarter minutes, allowing one minute thirty seconds and two minutes for operating, and during which time all the roots and teeth of the upper jaw were extracted.

In these prolonged cases the recovery is not quite so rapid as in the single dose cases, being more in the nature of the recovery from the major anæsthetics, though certainly more rapid.

One patient alone showed signs of collapse, probably due to the great number of teeth extracted.

I feel certain that with more courage in prolonging the anæsthesia the whole of the mouth could be cleared of teeth and the patient thoroughly recovered in probably less than five minutes from the commencement of administration, allowing the patient to leave the operating-room in less than ten minutes. I need hardly point out what a boon this would be.

Dr. Rolland has maintained complete anæsthesia for an operation lasting one hour, and has himself been anæsthetized over one hundred times, and on one occasion was kept under its influence for twenty-five minutes whilst observations were recorded.

The question most naturally occurring to all is whether somnoforme possesses dangers like chloroform, etc. In answer to this

Dr. Rolland states that if you deprive any patient of the essential amount of oxygen necessary to life any anæsthetic will produce death. If somnoforme is properly administered, death should not ensue, its great safety lying in the fact of its rapid elimination.

Another question is likely to be asked by many dentists,—Is it possible for a dentist to both administer somnoforme and operate himself, as many do with nitrous oxide?

Yes! I have done so for experiment, but only in one-dose cases. I do not think it advisable with this or any other anæsthetic. It is the work of the anæsthetist to look after the patient, and the operator to devote himself entirely to operating; the object should be to allow the patient to recover in as short a time as possible.

In conjunction with Professor Sherrington, University College, somnoforme was administered to several animals, giving much the same results as observed in the human subject. The analgesic condition was extremely well marked and tested. A marked progressive effect was noticed in some of the animals.

It was proved by an experiment that death would supervene if the mask was held on the face too long, whether from intoxication of the drug or asphyxia we are unable to prove. Respiration was arrested first, the heart continuing to beat for many seconds afterwards.

After almost five minutes from the time the respirations of a cat ceased (heart still beating), it was brought round to perfect health by artificial respiration.

As compared with other anæsthetics, the most remarkable feature was undoubtedly the rapid anæsthetization and the subsequent rapid recovery.—*The Dental Record.*

